# PLEK: a tool for predicting long non-coding RNAs and messenger RNAs based on an improved *k-*mer scheme

**DOI:** 10.1186/1471-2105-15-311

**Published:** 2014-09-19

**Authors:** Aimin Li, Junying Zhang, Zhongyin Zhou

**Affiliations:** School of Computer Science and Technology, Xidian University, Xi’an, PR China; School of Computer Science and Engineering, Xi’an University of Technology, Xi’an, PR China; Department of Molecular and Cell Biology, School of Life Sciences, University of Science and Technology of China, Hefei, PR China; State Key Laboratory of Genetic Resources and Evolution, Kunming Institute of Zoology, Chinese Academy of Sciences, Kunming, PR China

**Keywords:** RNA-seq, lncRNA, *k*-mer, Prediction, *de novo* sequencing, *de novo* assemble

## Abstract

**Background:**

High-throughput transcriptome sequencing (RNA-seq) technology promises to discover novel protein-coding and non-coding transcripts, particularly the identification of long non-coding RNAs (lncRNAs) from *de novo* sequencing data. This requires tools that are not restricted by prior gene annotations, genomic sequences and high-quality sequencing.

**Results:**

We present an alignment-free tool called PLEK (***p***redictor of ***l***ong non-coding RNAs and m***e***ssenger RNAs based on an improved ***k***-mer scheme), which uses a computational pipeline based on an improved *k*-mer scheme and a support vector machine (SVM) algorithm to distinguish lncRNAs from messenger RNAs (mRNAs), in the absence of genomic sequences or annotations. The performance of PLEK was evaluated on well-annotated mRNA and lncRNA transcripts. 10-fold cross-validation tests on human RefSeq mRNAs and GENCODE lncRNAs indicated that our tool could achieve accuracy of up to 95.6%. We demonstrated the utility of PLEK on transcripts from other vertebrates using the model built from human datasets. PLEK attained >90% accuracy on most of these datasets. PLEK also performed well using a simulated dataset and two real *de novo* assembled transcriptome datasets (sequenced by PacBio and 454 platforms) with relatively high indel sequencing errors. In addition, PLEK is approximately eightfold faster than a newly developed alignment-free tool, named Coding-Non-Coding Index (CNCI), and 244 times faster than the most popular alignment-based tool, Coding Potential Calculator (CPC), in a single-threading running manner.

**Conclusions:**

PLEK is an efficient alignment-free computational tool to distinguish lncRNAs from mRNAs in RNA-seq transcriptomes of species lacking reference genomes. PLEK is especially suitable for PacBio or 454 sequencing data and large-scale transcriptome data. Its open-source software can be freely downloaded from https://sourceforge.net/projects/plek/files/.

**Electronic supplementary material:**

The online version of this article (doi:10.1186/1471-2105-15-311) contains supplementary material, which is available to authorized users.

## Background

With the development of high-throughput transcriptome sequencing techniques (RNA-seq) [1], numerous transcripts have been identified in many species [2–4]. A new class of transcripts, long non-coding RNAs (lncRNAs, typically >200 nt), are of particular interest because they contribute to many important biological processes, such as dosage compensation [5], regulation of gene expression [6] and cell cycle regulation [7, 8]. Moreover, a number of studies showed that mutations and dysregulations in lncRNA genes were associated with human diseases [9, 10], such as cancers [11–13]. It remains a challenge to distinguish mRNAs from lncRNAs, especially for *de novo* sequencing without highly confident reference sequences and comprehensive annotations, because lncRNAs show many features similar to mRNAs, such as poly(A) tails, splicing and approximate sequence length [14]. Until now, several tools, such as CPC [15] and PhyloCSF [16], have been developed based on known protein databases, intrinsic sequence features and sequence conservation properties. These tools show varied efficiencies in distinguishing lncRNAs from mRNAs for different datasets. Most of them rely heavily on sequence alignment, and are therefore time-consuming and restricted by prior annotations. Recently, a software tool named Coding-Non-Coding Index (CNCI) was developed [17]. It discriminates coding from non-coding transcripts using intrinsic sequence features. CNCI performs better than previous tools for poorly annotated species or those without whole-genome sequence information, but it may misclassify transcripts when there are insertion or deletion (indel) sequencing errors among them. Such errors are common in the current Roche (454) and Pacific Biosciences (PacBio) sequencing platforms [18–21].

In this study, we developed a characteristic *k*-mer based alignment-free tool named PLEK, to solve the above-mentioned problems. PLEK takes calibrated *k*-mer frequencies of a transcript sequence as its computational features. With these features, the support vector machine (SVM) algorithm was used to build a binary classification model to separate lncRNAs from mRNAs. The classification model achieved high accuracy (95.6%) on training data with 10-fold cross-validation. PLEK also performed well on data from other vertebrates, using the classification model built from human training data. For transcripts containing indel sequencing errors, PLEK also attained high accuracy (>94%) in simulated and real transcriptome datasets. Moreover, PLEK is 8 times faster than CNCI and 244 times faster than CPC on the same test data. Therefore, PLEK is an accurate, robust and fast tool. It is suitable for vertebrates lacking high-quality genome sequences and annotation information, and is especially effective for the *de novo* assembled transcriptome data generated by PacBio or 454 sequencing platforms.

## Implementation

Careful selection of high-quality training data and their appropriate computational features is crucial to build an accurate, robust and fast binary classifier. In this section, we describe the data used to build the classification model and to test its performance. We then describe the distinct computational pipeline of *k*-mer usage. This is followed by the construction of a binary classifier using these data, *k*-mer usage features and SVM algorithm. Finally, we introduce simulation of indel sequencing errors on human protein-coding transcripts and transcriptome sequencing data from PacBio and 454 platforms.

### Data description

The RefSeq [22, 23] and GENCODE [24–26] projects provide comprehensive, non-redundant and well-annotated set of sequences, which can be used to build high-quality training and test datasets. Human protein-coding transcripts were downloaded from the RefSeq database (release 60) and human long non-coding transcripts were collected from GENCODE v17. There were 34,691 protein-coding transcripts with the length of >200 nt in the human RefSeq dataset, and 22,389 long (>200 nt) non-coding transcripts in the human GENCODE dataset. For performance assessment of cross-species prediction, we gathered transcripts from other vertebrates from the Ensembl [27] database (v72) (Table 1). To compare PLEK against CNCI, CPC and PhyloCSF, 6,015 mouse lncRNAs were gathered from GENCODE database (vM2). Mouse mRNAs were collected from RefSeq (release 60) and those with ‘putative’, ‘predicted’ or ‘pseudogene’ annotations were excluded. All the sequences used were longer than 200 nt.

**Table 1:** **Data sources and performance of cross-species prediction**

Species	Data source	Number of transcripts	Accuracy of CNCI	Accuracy of PLEK
*Mus musculus*	RefSeq mRNA	26062	**93.9%**	88.1%
	Ensembl ncRNA	2963	**97.1%**	89.9%
*Danio rerio*	RefSeq mRNA	14493	**95.3%**	91.3%
	Ensembl ncRNA	419	89.3%	**90.9%**
*Xenopus tropicalis*	RefSeq mRNA	8874	92.9%	**94.5%**
	Ensembl ncRNA	279^*^	99.7%	**100.0%**
*Bos taurus*	RefSeq mRNA	13190	94.3%	**94.8%**
	Ensembl ncRNA	182	**100.0%**	99.5%
*Pan troglodytes*	RefSeq mRNA	1906	**90.2%**	87.1%
	Ensembl ncRNA	1166	**100.0%**	99.9%
*Sus scrofa*	RefSeq mRNA	3978	**93.4%**	85.1%
	Ensembl ncRNA	241	95.9%	**98.3%**
*Macaca mulatta*	RefSeq mRNA	5709	**92.0%**	85.0%
	Ensembl ncRNA	359	99.7%	**100.0%**
*Gorilla gorilla*	RefSeq mRNA	33025	**87.4%**	83.8%
	Ensembl ncRNA	367	**99.7%**	**99.7%**
*Pongo abelii*	RefSeq mRNA	3401	93.4%	**98.0%**
	Ensembl ncRNA	392	99.8%	**100.0%**

PLEK and CNCI were tested on the same data; better accuracies are shown in bold face type. For RefSeq mRNAs, those with ‘putative’, ‘predicted’ or ‘pseudogene’ annotations were excluded (except for *Gorilla gorilla*).

^*^279 non-coding transcripts with lengths of more than 150 nt.

### Improved *k-*mer scheme

To characterize lncRNA and mRNA transcript sequences, we used *k*-mer usage and sliding-windows with a one-nucleotide step-length to analyze each transcript. A *k*-mer pattern is a specific string with *k* nucleotides, each can be *A*, *C*, *G* or *T*. For *k =* 1 to 5, we had 4 + 16 + 64 + 256 + 1024 = 1,364 patterns: 4 *one*-mer patterns, 16 *two*-mer patterns, 64 *three*-mer patterns, 256 *four*-mer patterns, and 1,024 *five*-mer patterns.

In designing a sliding-window of length *k, k = 1,2,…,5*, which slides along the transcript of length *l* by a step-length of one nucleotide, if the string in the transcript within the window matched with some pattern *i*, the occurrence number of the pattern in the transcript, denoted by *c*_*i*_, was increased by one. We did not simply use usage frequency *c*_*i*_*/s*_*k*_, *i =* 1 to 1364 (where *s*_*k*_ was the total number of times that the sliding-window of size *k*-nt could slide along the transcript, *s*_*k*_*= l-k* + 1); however, we calibrated it as *f*_*i*_ by a factor relating to the length of the pattern, *w*_*k*_, as the features of the transcript for prediction. The features used for prediction were given in formula (1).
123

### Construction of classification model

To produce a balanced training dataset, we collected all the 22,389 long non-coding transcripts from the GENCODE v17 dataset (labelled as the “negative” class) and randomly selected 22,389 protein-coding transcripts from the human RefSeq dataset (labelled as the “positive” class). The 1,364 calibrated *k*-mer usage frequencies of each transcript were regarded as computation features. First, these calibrated frequencies were normalized to the range from 0 to 1 using the *svm-scale* program from the LIBSVM package (version 3.17) [28]. Second, a support vector machine (SVM) with a radial basis functional kernel, whose variance is *gamma*, was selected as the binary classifier. The optimal *C* of the SVM and *gamma* of the kernel were obtained using the *grid.py* script of the LIBSVM package. During the process of parameter searching, 10-fold cross-validation was carried out to assess the performance of the classification model for each *C* and *gamma* parameter. Finally, we built an SVM binary classifier with the optimal *C* and *gamma*.

### Simulation of indel sequencing errors

Assembly of transcripts is made difficult by short read length sequences, which are typically generated by next-generation sequencing technologies [29, 30], especially by Illumina sequencing platforms. In contrast, PacBio and 454 platforms generate longer reads, which tend to be more easily assembled than short reads [31]. A large number of studies have been performed on these two kinds of sequencers [32–38]. However, the indel sequencing error rates are relatively high in PacBio and 454 sequencing data [19, 39]. A tool robust to such errors is desirable to distinguish lncRNAs and mRNAs, and facilitates annotation of lncRNAs and mRNAs of a species without whole-genome sequences.

We simulated single-base homopolymer-associated indel sequencing errors in protein-coding transcripts to evaluate the robustness of our tool, because they are the most typical indel sequencing errors in PacBio and 454 sequencing platforms. Without loss of generality, we simulated 0 to 3 single-base indel sequencing errors per 100 bases (the error rate *p* was 0% to 3%). For a transcript with a length of *l* bases, it had *n = lp* indel errors. We first counted the number of homopolymers of various lengths. Suppose the corresponding number of different homopolymers with lengths of *l*_*1*_*, l*_*2*_*, …, l*_*t*_ was *m*_*1*_*, m*_*2*_*, …, m*_*t*_, respectively, where *l*_*1*_*> l*_*2*_*> … > l*_*t*_, and *t* is the number of different lengths of homopolymers. A biological fact is that the likelihood of an indel error increases with the length of a homopolymer [19], even with no possibility that the indel error is in place between homopolymers. Thus, the indel errors start with the longest homopolymers with the length of *l*_*1*_. If *m*_*1*_*< n*, the homopolymers with the length of *l*_*2*_ are also inserted or deleted with bases, and so on, until *n* relatively longer homopolymers are processed. For these *n* homopolymers, we randomly inserted or deleted an identical base. If there are many homopolymers and few indels, the positions of indels will be evenly distributed in the transcripts (see Additional file 1 for an example).

### Construction of a real sequencing dataset

We used following two transcriptome datasets, sequenced by PacBio and 454 platforms, to test the performance of PLEK on *de novo* assembled transcripts without reference genomes. The first dataset was recently released by PacBio (Pacific Biosciences, Menlo Park, CA, USA). After full-length cDNA sequencing of the human MCF-7 transcriptome by the PacBio Isoform Sequencing (Iso-Seq) technology, PacBio used an iterative clustering algorithm and Quiver [40] to assemble *de novo* 44,531 polished, full-length and non-redundant transcripts. We aligned these transcripts with human RefSeq mRNAs (release 60) and GENCODE lncRNAs (v17) using NCBI Blastn with the parameters: *-task megablast -evalue 1e-10 -perc_identity 80 -max_target_seqs 1*. Then, filtering these transcripts by query coverage >80%, subject coverage >80% and gaps >0, we obtained 3,306 transcripts (3,185 mRNAs and 121 lncRNAs) that may have had indel sequencing errors.

The second dataset, a HelaS3 cell line transcriptome, was sequenced by a 454 GS FLX Titanium platform and is available at the Sequence Read Archive (SRA) under accession no. SRA063146 [41]. LUCY (1.20p) [42] and SeqClean (ftp://occams.dfci.harvard.edu/pub/bio/tgi/software/) were used to discard the adapter sequences, low quality reads and sequences of less than 50 bp, resulting in 4,222,133 high-quality sequences. Trinity r20131110 [43] with default parameters was used to assemble these *de novo.* Trinity assembled 65,583 transcripts, which were subsequently aligned with human RefSeq mRNAs and GENCODE lncRNAs, and were filtered in the same manner as the first dataset. Finally, 3,098 transcripts with possible indel sequencing errors were retained (3,045 mRNAs, 53 lncRNAs).

### Running PhyloCSF on mouse datasets

In order to compare PLEK against PhyloCSF [16] on mouse datasets, we set up a local instance of Galaxy [44]. Multiple alignments of 59 assemblies to the mouse genome (mm10/GRCm38) were downloaded from the UCSC Genome Browser (http://hgdownload-test.sdsc.edu/goldenPath/mm10/multiz60way/maf/). BED files describing mouse lncRNA and mRNA transcripts were loaded onto the local Galaxy webserver and the tool ‘Stitch Gene Blocks’ was used to retrieve multiple alignment files with sequence entries for the following genome builds based on the 60-way Multiz alignment to *mm10*: *mm10*, *rn5*, *dipOrd1*, *cavPor3*, *speTri2*, *oryCun2* and *ochPri2*. Genome build names were converted to common names and PhyloCSF was run using the options: *--frames = 3, --orf = StopStop3* and *--removeRefGaps*.

## Results

### Different usage frequencies of *k-*mer strings

To verify the difference between mRNA and lncRNA in *k*-mer string usage, we calculated the calibrated usage frequencies of all the 1,364 *k*-mer patterns in the positive training dataset (22,389 protein-coding transcripts) and negative training dataset (22,389 long non-coding transcripts). We used the *Wilcox* rank-sum test to determine which *k*-mer pattern usage was significantly different between mRNAs and lncRNAs. With a significance level of 10^-6^, we found that 1,278 patterns were significantly different in their usage (see Additional files 2 and 3). This demonstrated that the differences between the usage frequencies of these *k*-mers could largely differentiate the two groups. Therefore, our improved *k*-mer scheme is a suitable algorithm to distinguish mRNAs and lncRNAs. 10-fold cross-validation on the human training datasets was performed. The accuracy was 95.6%. Although PLEK was not better than the state-of-the-art CNCI tool on the dataset (CNCI’s accuracy was 97.3% on human RefSeq and GENCODE datasets), it worked better on transcriptome data from PacBio and 454 datasets (see section entitled ‘Performance on PacBio and 454 datasets’).

### Performance in cross-species prediction

At present, genomic sequences and annotations of most organisms are of poor quality or are unavailable. To analyze the transcriptome data of these organisms, we could draw wide support from the well-annotated related organisms in a cross-species manner. For example, we could try using the models built by human training data to analyze data from other vertebrates that have not been deeply explored.

We tested PLEK on several other vertebrates to assess its performance in cross-species prediction of protein-coding and non-coding transcripts, and found that it worked well (Table 1). This result demonstrated that PLEK exhibits good performance in cross-species prediction, as the CNCI does, which performed uniformly on all the species of vertebrates [17]. We also found that the more similar the genome of a vertebrate was to that of human, the better the performance of the model. Therefore, for species without reference genomes or with poor annotation information, one could use transcripts and annotation of closely related organisms to build models to distinguish their protein-coding and non-coding transcripts.

### Robustness to indel sequencing errors

**Figure 1:**
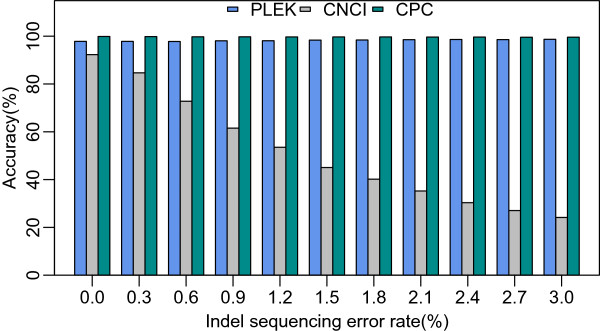
**Comparison of robustness towards indel sequencing errors.** The x-axis is the indel numbers per 100 bases (indel sequencing error rates). Performance (accuracy) of CNCI declines significantly as the indel error rate increases.

We applied PLEK to human protein-coding transcripts with simulated indel sequencing errors to evaluate its robustness and compare its performance with that of CPC and CNCI. CPC is widely used to assess the protein-coding potential of a transcript based on alignment with a protein database [14, 45, 46]. CNCI effectively distinguishes protein-coding and non-coding sequences independent of reference genomes and known annotations by profiling adjoining nucleotide triplets (ANT). CNCI provides highly accurate prediction of transcripts assembled from RNA-seq data in a cross-species manner. In our study, human protein-coding transcripts were extracted from the RefSeq database and the overlapping transcripts with the training set were removed. Using the indel error simulation approach, we examined robustness performance across different indel sequencing error rates from 0% to 3% (Figure 1). The results showed that the accuracies of PLEK and CPC were not affected by a small amount of indels, whereas the accuracy of CNCI decreased significantly with increasing indel error rates, indicating that PLEK is a robust tool for distinguishing protein-coding and non-coding transcripts with homopolymer-associated indel sequencing errors.

### Performance on PacBio and 454 datasets

We compared the performance of PLEK with that of CNCI and CPC using *de novo* assembled transcripts derived from PacBio and 454 platforms. 3,306 transcripts from the MCF-7 transcriptome sequenced by a PacBio platform and 3,098 transcripts from HelaS3 transcriptome generated by a 454 platform were fed into these three tools, respectively. PLEK maintained its high accuracy, 0.947 on the PacBio dataset and 0.954 on the 454 dataset, which was higher than that of CNCI (0.913 and 0.937, respectively) (Table 2). CPC achieved the highest accuracy (>0.970), but the lowest specificity (<0.472). CPC remained high positive and negative predictive values (PPV and NPV) (>0.926). PLEK and CNCI had relatively better PPV (>0.991) but poor NPV (<0.407). Sensitivity, specificity, PPV, NPV and accuracy were calculated using the following formulae:


*FN*, false negative; *FP*, false positive; *TN*, true negative; *TP*, true positive.

**Table 2:** **Performances on transcripts derived from PacBio and 454**

Dataset	Tool	Sensitivity	Specificity	PPV	NPV	Accuracy
MCF-7 (PacBio)	PLEK	0.947	**0.958**	**0.998**	0.407	0.947
	CPC	**0.999**	*0.190*	0.970	**0.958**	**0.970**
	CNCI	0.918	0.787	0.991	*0.269*	0.913
HelaS3 (454)	PLEK	0.955	**0.925**	**0.999**	0.262	0.954
	CPC	**0.999**	*0.472*	0.991	**0.926**	**0.990**
	CNCI	0.939	0.811	0.997	*0.189*	0.937

Bold face type indicates the best performances (sensitivity, specificity, PPV, NPV, accuracy) among PLEK, CPC and CNCI. Italic face type indicates the worst specificity and NPV among these tools.

### Performance comparison on mouse datasets

**Figure 2:**
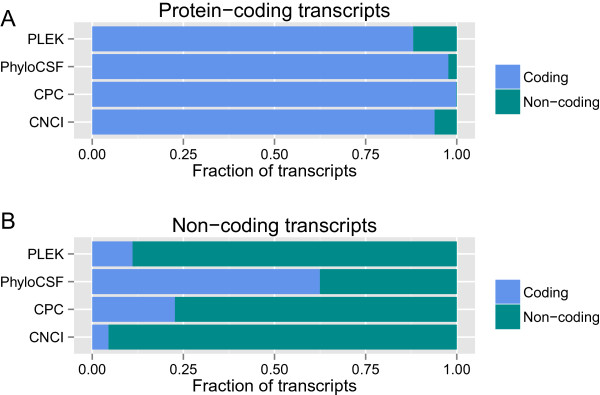
**Results of PLEK, CPC, CNCI and PhyloCSF on mouse datasets. (A)** The fraction of protein-coding transcripts classified as coding or non-coding. **(B)** The fraction of non-coding transcripts classified as coding or non-coding. Data were collected from RefSeq mouse protein-coding transcripts (release 60) and GENCODE mouse long non-coding transcripts (vM2). Shown is the fraction of transcripts classified as coding or non-coding by each tool. All these tools performed well on protein-coding transcripts. PLEK and CNCI outperformed CPC and PhyloCSF on long non-coding transcripts.

We compared the performance of PLEK with that of CNCI, CPC and PhyloCSF on the mouse datasets which were composed of 6,015 lncRNAs and a random sample of 6,015 mRNAs. We evaluated these four tools and measured their accuracy on protein-coding and long non-coding transcripts, respectively. Figure 2 shows the fraction of transcripts that were classified as coding or non-coding by each tool. On protein-coding transcripts, all these tools performed well. Only a small fraction of protein-coding transcripts were misclassified as non-coding (Figure 2A). On non-coding transcripts, PLEK and CNCI outperformed PhyloCSF and CPC. At least 22% non-coding transcripts were misclassified as coding by PhyloCSF and CPC (Figure 2B). These results indicate that PLEK is a reasonably efficient tool.

### Computational performance

Advances in sequencing technologies have produced huge amount of transcriptome data. To date (Mar 17, 2014), there are approximately 4,158 studies of transcriptome analysis in the Sequence Read Archive (SRA). 1,606 of these studies generated more than 10 Gb bases, and 234 of them produced more than 100 Gb bases. The scale of such data will become much larger when we comprehensively analyze the RNA-seq data from different studies. Thus it is necessary to develop a high-speed tool to separate lncRNAs from mRNAs in large-scale transcriptome data.

We measured computation time of PLEK, CNCI, CPC and PhyloCSF on a sample of 1,000 protein-coding sequences and 1,000 long non-coding sequences randomly selected from human RefSeq and GENCODE v17 databases, respectively. All these tools were run in a single-threading manner on an IBM X3650 M4 computer equipped with two E5-2640 CPUs and 64 GB of RAM. PLEK took 128 seconds to process the data, which was approximately eightfold faster than CNCI (1,048 seconds), 244 times than CPC (31,247 seconds) and 1,421 times than PhyloCSF (181,925 seconds) (Table 3). Additionally, PLEK could be easily configured for multi-threading parallel computing, which will further save computation time. Thus, PLEK is especially suitable for classifying a large number of transcripts conducted by RNA-seq technologies.

**Table 3:** **Comparison of computational performances of PLEK, CNCI, CPC and PhyloCSF**

Performance	PLEK	CNCI	CPC	PhyloCSF
Run time^a^ (seconds)	128	1048	31247	181925^e^
Multi-threading^b^	Yes	Yes	No^d^	No
Online running^c^	No	No	Yes	No

Computational time was tested on 1,000 human mRNA transcripts and 1,000 human lncRNA transcripts.

^a^Computation time consumed when run in a single-threading manner.

^b^Can the software tool run in a multi-threading manner?

^c^Does the software tool provide a website for users to run online?

^d^CPC improves its computational performance using a page-cache method on its website.

^e^Bed files were load onto the Galaxy webserver (http://galaxy.nbic.nl/) and the tool ‘Stitch Gene Blocks’ was used to retrieve multiple alignment files with sequence entries for the following genome builds based on the 10-way Multiz alignment to *hg19*: *hg19*, *panTro2*, *tarSyr1*, *micMur1*, *otoGar1* and *rheMac2*. PhyloCSF was run using the options: *--removeRefGaps*.

## Discussion

Several studies have identified numerous lncRNAs from RNA-seq data [43, 45, 47–51]. However, the transcriptomes of many species, with partial or missing reference genomes, have been studied using PacBio or 454 sequencing techniques [32–38]. PacBio and 454 platforms generate longer read lengths than Illumina, which make it easier to assemble *de novo* transcripts without reference genomes [31, 38]; however, transcripts generated by these platforms have relatively higher indel sequencing error rates [19, 39]. Furthermore, an increasing number of RNA-seq data have been generated and the data scale is expanding rapidly with advances in high-throughput sequencing technologies. Therefore, it is necessary to develop a tool independent of known annotations and suitable for cross-species prediction that is robust to indel sequencing errors and fast enough to be affordable for large-scale data.

Appropriate computational features are very important for classification. Although conventional *k*-mer feature has been employed in several studies, such as gene identification [52], piRNA prediction [53], class-specific motif detection [54] and miRNA classification [55], we found that the proposed improved *k*-mer usage frequencies were good features to identify lncRNAs.

**Figure 3:**
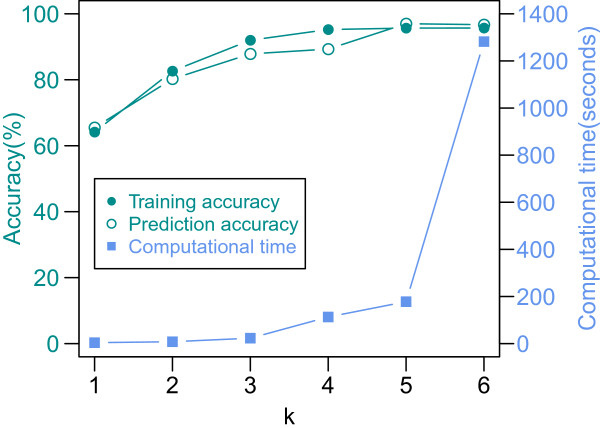
**Performance comparison of various ranges of** ***k.*** On the x-axis, ‘5’ means that *k* ranged from 1 to 5. Training data comprised 22,389 human RefSeq mRNA transcripts and 22,389 GENCODE lncRNA transcripts. SVM classifiers were trained using 10-fold cross-validation on the training datasets. The figure indicates that the computation load rises and the accuracy increases along as *k* increases.

More features would be used for prediction with increasing *k*. For example, 340 features are used when *k* ranges from 1 to 4, 1,364 features when *k* ranges from 1 to 5, and so on. Prediction accuracy increases with the increasing *k*; however, this is accompanied by an increasing computation load (Figure 3). As we increased *k*, and thus added more sequence features, we were able to better discriminate between lncRNA and mRNA sequences. *K-*mers with lengths greater than five do not significantly change the discrimination power of the SVM. Thus, for a trade-off between computational time and accuracy, we determined the range of *k* as 1 to 5. The model, PLEK, which uses these features, attained high prediction accuracy in 10-fold cross-validation on a human training dataset. PLEK runs faster than previous tools, CPC and CNCI, because CPC is an alignment-based tool and CNCI is obliged to calculate Hexamer (*k =* 6) usage.

We multiplied the *k*-mer usage frequencies by various calibration factors (formula 3), which gave more weightage to those *k*-mer strings that are longer in length. Intuitively*, k*-mer usage frequencies generally decrease with the increase of the size of *k*-mer strings. For instance, the frequency of nucleotide *G* is theoretically around 0.25, while that of *GGGGG* is about 0.001. However, longer *k*-mer strings may contain more information than shorter ones: several studies indirectly use Hexamer and ANT (*k =* 6) usage as features to classify coding from non-coding RNAs [17, 56]. We multiplied usage frequencies of longer *k*-mer strings by relatively larger factors for calibration, hence outweighing longer *k*-mer strings, and thereby improving the weights of the corresponding features.

The step-lengths of sliding-windows in a previous tool, CNCI, are fixed at three [17, 53]. A single-base indel sequencing error in a protein-coding sequence may result in a false shift in reading frame [57], which can dramatically affect the performance of CNCI (Figure 1). In contrast, PLEK uses a sliding-window of size *k*, where *k* ranges from 1 to 5, to slide along a nucleotide sequence from its 5′ to 3′ end to count the occurrence number of *k*-mer strings. Frameshifts do not exert a strong influence on the calculation of *k*-mer usage when using a sliding-window of one-nucleotide step-length. This is why the results showed that our approach deals with indel sequencing errors robustly. This feature was also confirmed by the test on the real PacBio and 454 datasets (Table 2). On these datasets, the specificity of PLEK was the highest among these tools. Moreover, PLEK achieved an optimal balance between high specificity and high sensitivity (0.946 and 0.942 on PacBio, 0.955 and 0.925 on 454). PLEK thoroughly outperformed CNCI. Although CPC produced the highest sensitivity, it suffered from poor specificity (0.190 on PacBio, 0.472 on 454). CPC was more likely to misclassify lncRNAs as mRNAs, with high false-positive rates. There were large amount of mRNAs (96.3% in PacBio and 98.3% in 454) and few lncRNAs (3.7% in PacBio and 1.7% in 454) in these datasets, in conjunction with CPC’s high sensitivity, which led to the high accuracy of CPC on the test data.

Compared with CPC, the sensitivity and specificity of PLEK is well balanced (Table 2). On the real mouse datasets including the same number of mRNA and lncRNA transcripts, PLEK could obtain high PPV and NPV (Figure 2). However, NPV of PLEK is probably low on highly imbalanced datasets or organisms with compact genomes. The number of coding RNAs is at least two orders of magnitude greater than that of non-coding RNAs on the real PacBio and 454 datasets we used in this study. In this situation, as most of the transcripts are coding, the prediction value over the transcripts called as non-coding is very low. Even quite a small portion, say 1%, of misclassification of mRNAs is likely to result in remarkable decrease of NPV (Table 2). PLEK produces a decision value for each transcript and then labels it according to this criterion: the cut-off of discriminating transcripts is 0 by default, those with >0 decision values are labelled as mRNAs, and <0 as lncRNAs. Generally, greater absolute decision values indicate greater confidence in the prediction. Therefore, we can apply various criteria in different conditions to achieve satisfactory performance (high PPV or NPV). Similar criteria were also used or established in several researches using CPC and PhyloCSF [49, 51, 58]. Another method of transcending this limitation of PLEK is to perform additional analyses, such as orthology analyses of the ORFs contained in transcripts, scanning for known protein domains, etc. [45, 49, 58].

The classifier we currently released on Sourceforge was built on human training data. Although it obtained well performance on most vertebrates (Table 1), it might not be directly suitable to other species with very different sequence composition to human, such as *Drosophila melanogaster*, *Caenorhabditis elegans* and *Arabidopsis thaliana*. Therefore, we provided a Python script, named ‘PLEKModelling.py’, to assist users to build new classifiers. For the species that have not been well-explored, no sufficient reliable lncRNA transcripts are now available to build classifiers in most cases. We encourage users to use as many reliable transcripts of their relatives as possible to build classifiers. For instance, we trained a classifier using *Zea mays* Ensembl mRNA transcripts and lncRNA transcripts identified by Li *et al.* [59], and this classification model performed well on *Arabidopsis thaliana* and *Oryza sativa* datasets (Additional file 4).

Therefore, PLEK is a valuable alignment-free tool, which is accurate, robust and fast, to identify lncRNAs in *de novo* assembled transcripts without reference genomes.

## Conclusions

Long non-coding RNAs are receiving increasing attention, and distinguishing lncRNAs from mRNAs in *de novo* sequencing data without reference genomes represents a challenge. To solve this problem, we designed an alignment-free tool, PLEK, which is based on an improved *k*-mer scheme. The computation of *k*-mer usage is different from that used in previous studies [53, 55]: the step-lengths of the sliding-windows are one nucleotide, and the *k*-mer usage is calibrated according to the size of *k*-mer strings. PLEK worked well on human training data and in a cross-species manner on other vertebrates using the model built from human training data. It also performed well on simulated transcripts and real *de novo* assembled PacBio and 454 transcriptomes, all of which include relatively high levels of indel sequencing errors than data generated by Illumina platforms. PLEK struck a better balance between high specificity and high sensitivity than CPC on PacBio and 454 sequencing data. In addition, PLEK runs at least eightfold faster than previous available tools, CPC and CNCI. The results demonstrated that PLEK is particularly suited to transcriptome data with indel sequencing errors and growing large-scale transcriptome datasets. Thus, PLEK is a useful tool for distinguishing protein-coding and non-coding sequences from high-throughput sequencing data of many species without reference genomes.

## Availability and requirements

**Project name:** PLEK.

**Project home page:**https://sourceforge.net/projects/plek/ website.

**Operating systems(s):** Linux.

**Programming language:** C, Python.

**Other requirements:** gcc, g++, Python.

**License:** GNU Public License version 3 (GPLv3).

**Any restrictions to use by non-academics:** none.

## Electronic supplementary material

Additional file 1: **Demonstration of indel sequencing error simulation.** (PDF 160 KB)

Additional file 2: **Features and their P-values of rank-sum test.** (XLS 163 KB)

Additional file 3: **Visualization of the** ***k-*** **mer usage differences between mRNAs and lncRNAs.** (PDF 146 KB)

Additional file 4: **Building a new classifier using PLEKModelling.py.** (PDF 328 KB)

## Abbreviations

454: Roche 454
ANT: Adjoining nucleotide triplets
AUC: Area under the curve
bp: Base pair
CNCI: Coding-Non-Coding Index
CPC: Coding Potential Calculator
lncRNA: Long non-coding RNA
mRNA: Messenger RNA
NPV: Negative predictive value
nt: Nucleotide
PacBio: Pacific Biosciences
PLEK: Predictor of long non-coding RNAs and messenger RNAs based on an improved *k-*mer scheme
PPV: Positive predictive value
SVM: Support vector machine.
