# Supplementary material for: PLEK: a tool for predicting long non-coding RNAs and messenger RNAs based on an improved k-mer scheme
Source: BMC Bioinformatics. 2014 Sep 19;15(1):311. doi: 10.1186/1471-2105-15-311 (PMC4177586; doi:10.1186/1471-2105-15-311)
Supplement: Supplementary file 1 — Additional file 1: Demonstration of indel sequencing error simulation. (PDF 160 KB) [file 12859_2013_6586_MOESM1_ESM.pdf]

Additional file 1 of PLEK

Demonstration of indel sequencing error simulation

We take a protein-coding transcript *NM_001012425.1* from RefSeq for example. For convenience, we mark every homopolymer of longer than 3 bases as P_1, P_2, …, and so on (see **Box 1**). There are 18 homopolymers in total. We simulate 2 indels per 100 bases (indel error rate is 2%). For this transcript with a length of l=751 bases, it will have $n=l*p$=751*2%=15 indels if the indel error rate is p=2%. We first count the numbers of homopolymers of various lengths. The corresponding numbers of different homopolymers with lengths of l_1=6, l_2=5, l_3=4 are m_1=2, m_2=2, m_3=14, respectively, where $l_1 > l_2 > l_3$ (see **Table S1**)。The likelihood of an indel increases with the length of the homopolymer. The indels start with the longest homopolymers with the length of l_1=6. In other words, initially, there are indels at P_2 and P_{10} positions. Then, the homopolymers with the length of l_2=5 are also inserted or deleted with identical bases (at P_7 and P_{14} positions). Finally, 11 of 14 homopolymers of 4 bases are processed in the same way. For these homopolymers, we randomly insert or delete an identical base. If there are many homopolymers and few indels, the positions of indels will be evenly distributed in transcripts. See **Box 2**, insertions were marked as nucleotides with borders, and deletions with background shadow. The numbers in braces were the order in which we inserted or deleted identical bases.

Box 1 Homopolymers of longer than 3 bases in NM_001012425.1

Homopolymers of longer than 3 bases were marked as P_1, P_2, …, and so on. There are 18 homopolymers in total.

>gi|60218900|ref|NM_001012425.1| Homo sapiens chromosome 1 open reading frame 146 (C1orf146), mRNA;

ACCCTGAGCGGTCTCTGAGGACCTGGTGAGCAGATTGTTGCACCATTAGAAGCTAGGT

TGATCCACAGACAGATGGCTGAAAGTGGAAAAGAAAAAATAAAATGGACAACCACCA

P_1 P_2 P_3

TTATTATTAGCTCATCTCTTAAGAGTTATGAAGTTGCAACTGCCCTAGAAAATCGAAGCC

P_4

ACAAAGTTCGATATTCAGATTCAGTGGAAAATGGATCAATTATATTTTCTCTTTCTGGAG

P_5 P_6

TTGCATTTTTATTAATGGATACTAAGGAATGTCTTCTGTCAACTGAAGAAATATTTCTAG

P_7

CCAAAATTGAGAAATTTATTAACATTCACCAAAATAGTTTTTTGGTTTTGTCTGCTGCCC

P_8 P_9 P_{10} P_{11}

TCCATGGGCCTGAAGAATGGAAACTGATGTTCAGGATTCAGCAGAGATTCCTGGGTTG

TAACTTACGAATACTTCCAGTACACAACACAGTAAATGCTATTAATCTTATGTGCACTAT

AGCAAAGACTACCTCCAAACCATACATAGATAGCATTTGCTACAGAATGATAACAGCTA

AAGCTTACATCATTGAGCAAAGTCCTGTTTGGAAAACACTTCAGAAGATAAAACTGAA

P_{12} P_{13}

TAGTGATTCAGTTAACCCAAATTAGAGTACCAACTTAATGTTTTTCTCGAAGAATGTGA

P_{14} P_{15}

AAATAATTAGACCTGTTAAATTATAATATTCAAATATCTATTTAAAGACATTTATATTAATT

TGAAATAATAACATATACAATTAAAAGTGATTTTATTTTA

P_{16} P_{17} P_{18}

Table S1 Number of homopolymers in NM_001012425.1

The numbers of homopolymers of various lengths were counted. The corresponding numbers of different homopolymers with lengths of l_1=6, l_2=5, l_3=4 are m_1=2, m_2=2, m_3=14, respectively.

Homopolymer size	Homopolymer number	Position
l_1=6	m_1=2	P_2, P_{10}
l_2=5	m_2=2	P_7, P_{14}
l_3=4	m_3=14	P_1, P_3, P_4, P_5, P_6, P_8, P_9, P_{11}, P_{12}, P_{13}, P_{15}, P_{16}, P_{17}, P_{18}

Box 2 An example of homopolymer-associated indels in NM_001012425.1

Insertions were marked as nucleotides with borders, and deletions with background shadow. The numbers in braces were the order in which we inserted or deleted identical bases.

>gi|60218900|ref|NM_001012425.1| Homo sapiens chromosome 1 open reading frame 146 (C1orf146), mRNA;

ACCCTGAGCGGTCTCTGAGGACCTGGTGAGCAGATTGTTGCACCATTAGAAGCTAGGT

TGATCCACAGACAGATGGCTGAAAGTGGAAAAAGAAAAAATAAAAATGGACAACCACCA

P_1(5) P_2(1) P_3(6)

TTATTATTAGCTCATCTCTTAAGAGTTATGAAGTTGCAACTGCCCTAGAAAATCGAAGCC

P_4(7)

ACAAAGTTCGATATTCAGATTCAGTGGAAAATGGATCAATTATATTTTTCTCTTTCTGGAG

P_5 P_6(8)

TTGCATTTTTATTAATGGATACTAAGGAATGTCTTCTGTCAACTGAAGAAATATTTCTAG

P_7(3)

CCAAAAATTGAGAAATTTATTAACATTCACCAAAATAGTTTTTTTGGTTTTGTCTGCTGCCC

P_8(9) P_9(10) P_{10}(2) P_{11}

TCCATGGGCCTGAAGAATGGAAACTGATGTTCAGGATTCAGCAGAGATTCCTGGGTTG

TAACTTACGAATACTTCCAGTACACAACACAGTAAATGCTATTAATCTTATGTGCACTAT

AGCAAAGACTACCTCCAAACCATACATAGATAGCATTTGCTACAGAATGATAACAGCTA

AAGCTTACATCATTGAGCAAAGTCCTGTTTGGAAAACACTTCAGAAGATAAAAACTGAA

P_{12}(11) P_{13}(12)

TAGTGATTCAGTTAACCCAAATTAGAGTACCAACTTAATGTTTTTCTCGAAGAATGTGA

P_{14}(4) P_{15}(13)

AAAATAATTAGACCTGTTAAATTATAATATTCAAATATCTATTTAAAGACATTTATATTAATT

TGAAATAATAACATATACAATTAAAAGTGATTTTATTTTTA

P_{16} P_{17}(14) P_{18}(15)
