# Supplementary material for: PLEK: a tool for predicting long non-coding RNAs and messenger RNAs based on an improved k-mer scheme
Source: BMC Bioinformatics. 2014 Sep 19;15(1):311. doi: 10.1186/1471-2105-15-311 (PMC4177586; doi:10.1186/1471-2105-15-311)
Supplement: Supplementary file 3 — Additional file 3: Visualization of the k- mer usage differences between mRNAs and lncRNAs. (PDF 146 KB) [file 12859_2013_6586_MOESM3_ESM.pdf]

Additional file 3 of PLEK

Visualization of the *k*-mer usage differences between mRNAs and lncRNAs

To visualize the *k*-mer usage differences between mRNAs and lncRNAs, we define the log-ratio of the string usage frequencies between mRNAs and lncRNAs by formula (1), where a_m^+ is the average of the *m*-th feature in positive samples (*m*=1,2,…,1364). a_m^- is the average of the *m*-th feature in negative samples, r_m is the log-ratio (base 2) of positive samples' *k*-mer usage frequency average to negative samples' for the *m*-th feature.

$$r_m = \log(a_m^+ / a_m^-),\ m=1,2,\ldots,1364 \qquad (1)$$

The results are showed in **Figure S1**. Dark green lines (above 0) represent that these strings are more frequently used in mRNAs than lncRNAs, and blue lines (below 0), lncRNAs than mRNAs.

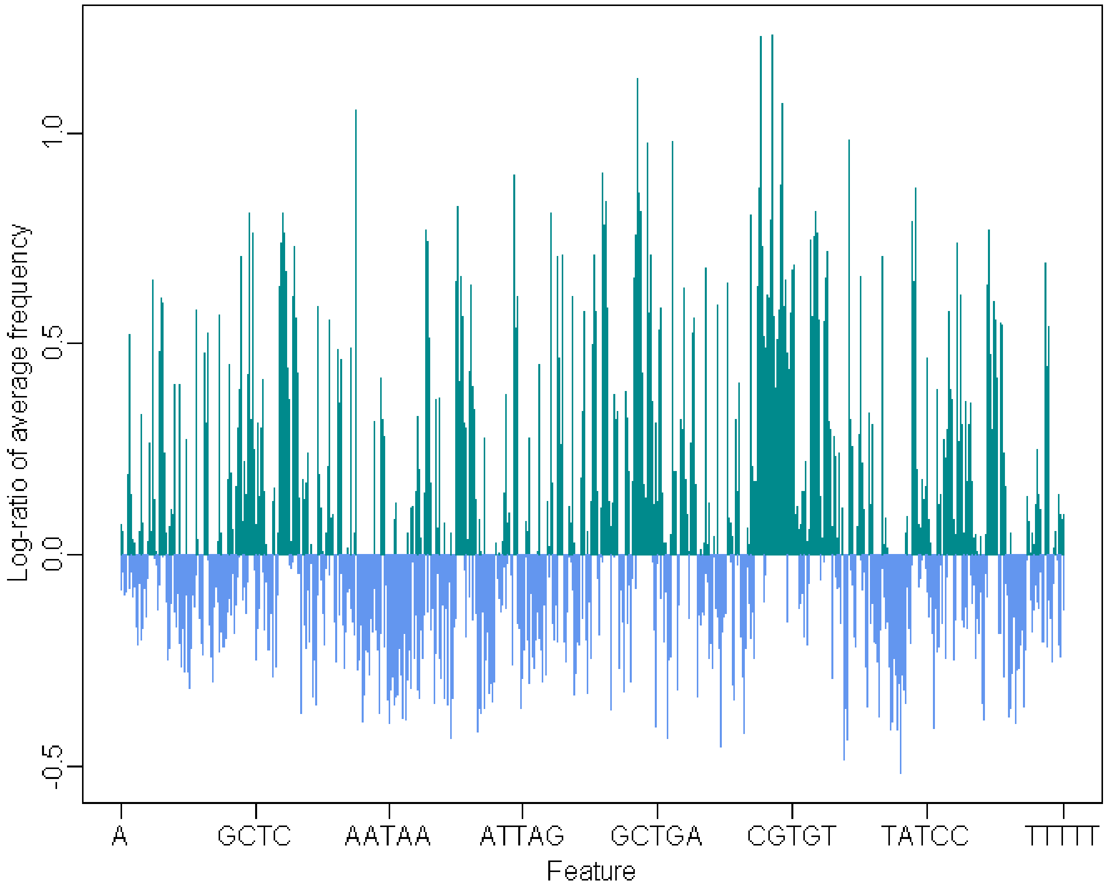

Figure S1 Average calibrated *k*-mer usage frequency log-ratio of protein-coding to non-coding transcripts.

The x-axis represents the *k*-mer strings (*A*, *C*, *G*, *T*, *AA*, *AC*, *AG*, *AT*, …, *TTTTT*). The y-axis represents the log-ratios of average calibrated *k*-mer usage frequencies in protein-coding transcripts to those in non-coding transcripts.
