# Supplementary material for: PLEK: a tool for predicting long non-coding RNAs and messenger RNAs based on an improved k-mer scheme
Source: BMC Bioinformatics. 2014 Sep 19;15(1):311. doi: 10.1186/1471-2105-15-311 (PMC4177586; doi:10.1186/1471-2105-15-311)
Supplement: Supplementary file 4 — Additional file 4: Building a new classifier using PLEKModelling.py. (PDF 328 KB) [file 12859_2013_6586_MOESM4_ESM.pdf]

Additional file 4 of PLEK

Building a new classifier using PLEKModelling.py

We trained a classifier using *Zea mays* Ensembl mRNA transcripts and lncRNA transcripts identified by Li *et al*. [1], and this classification model performed well on *Arabidopsis thaliana* and *Oryza sativa* datasets.

Data description

Maize protein-coding transcripts were gathered from Plant Ensembl (http://plants.ensembl.org/) release 17.

By exploiting available public EST databases, maize whole genome sequence annotation and RNA-seq datasets from 30 different experiments, Li *et al.* identified 20,163 putative maize lncRNAs [1]. These lncRNAs were annotated in GTF format and published on Genome Biology website. Some of them were annotated on unknown chromosomes, and strands of some lncRNAs are unknown. Such lncRNAs were excluded for our further analysis.

The training dataset we used was composed of 88,611 protein-coding transcripts and 13,799 lncRNA transcripts.

Building a classifier

We used the following Python script and command to automatically build a classifier:

```
python PLEKModelling.py   \
-mRNA     ensembl.Zea_mays.AGPv2.17.protein_coding.fa    \
-lncRNA   linli.genome_biology.maize.lncRNA.fa    \
-prefix     maize_ens_linli     \
-log2c     0,3,1     \
-log2g     -1,-5,-1     \
-thread   20
```

Where *ensembl.Zea_mays.AGPv2.17.protein_coding.fa* was the protein-coding transcripts; *linli.genome_biology.maize.lncRNA.fa* was the lncRNA transcripts; *maize_ens_linli* was the prefix of output files. The program ran in 20-threading runs and output a model file, *maize_ens_linli.model*, and a svm-scale range file, *maize_ens_linli.range*.

Users can also use the R script *PLEK_generate_scripts.R*, which is contained in PLEK 1.2, to generate shell script files, corresponding to various combinations of SVM *C* and *Gamma* parameters. And then submit these scripts to PBS/Torque to run in parallel.

Performance evaluation

We evaluated the performance of this new model only on protein-coding transcripts of several important model plants for long noncoding RNAs with detailed genomic structures are not available yet, or the number of them is too little to perform statistics.

We ran PLEK.py with this new model, *maize_ens_linli.model*, on *Arabidopsis thaliana* and *Oryza*

sativa protein-coding transcripts collected from Plant Ensembl (release 17). The format of command is as follows:

```
python PLEK.py  \
  -fasta      transcripts_in_fasta_file      \
  -out        plek_predicted_result   \
  -range      maize_ens_linli.range   \
  -model      maize_ens_linli.model
```

This model achieved high accuracy and the results were shown in Figure S1.

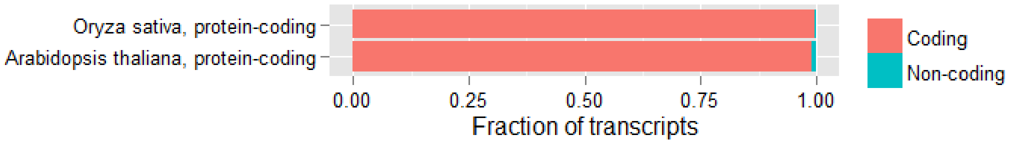

Figure S1 – Performance of maize model on two other plants. Shown is the fraction of transcripts classified as coding or non-coding for each set. PLEK achieved high accuracy on *Arabidopsis thaliana* and *Oryza sativa* protein-coding transcripts.

References

[1]. Li L, Eichten SR, Shimizu R, Petsch K, Yeh C-T, Wu W, Chettoor AM, Givan SA, Cole RA, Fowler JE: **Genome-wide discovery and characterization of maize long non-coding RNAs**. *Genome biology* 2014, **15**(2):R40.
